# PCGA: a comprehensive web server for phenotype-cell-gene association analysis

**DOI:** 10.1093/nar/gkac425

**Published:** 2022-05-26

**Authors:** Chao Xue, Lin Jiang, Miao Zhou, Qihan Long, Ying Chen, Xiangyi Li, Wenjie Peng, Qi Yang, Miaoxin Li

**Affiliations:** Program in Bioinformatics, Zhongshan School of Medicine and The Fifth Affiliated Hospital, Sun Yat-sen University, Guangzhou 510080, China; Research Center of Medical Sciences, Guangdong Provincial People's Hospital, Guangdong Academy of Medical Sciences, Guangzhou 510080, China; Program in Bioinformatics, Zhongshan School of Medicine and The Fifth Affiliated Hospital, Sun Yat-sen University, Guangzhou 510080, China; Program in Bioinformatics, Zhongshan School of Medicine and The Fifth Affiliated Hospital, Sun Yat-sen University, Guangzhou 510080, China; Program in Bioinformatics, Zhongshan School of Medicine and The Fifth Affiliated Hospital, Sun Yat-sen University, Guangzhou 510080, China; Program in Bioinformatics, Zhongshan School of Medicine and The Fifth Affiliated Hospital, Sun Yat-sen University, Guangzhou 510080, China; Program in Bioinformatics, Zhongshan School of Medicine and The Fifth Affiliated Hospital, Sun Yat-sen University, Guangzhou 510080, China; Program in Bioinformatics, Zhongshan School of Medicine and The Fifth Affiliated Hospital, Sun Yat-sen University, Guangzhou 510080, China; Program in Bioinformatics, Zhongshan School of Medicine and The Fifth Affiliated Hospital, Sun Yat-sen University, Guangzhou 510080, China; Key Laboratory of Tropical Disease Control (Sun Yat-sen University), Ministry of Education, Guangzhou 510080, China; Center for Precision Medicine, Sun Yat-sen University, Guangzhou 510080, China; Guangdong Provincial Key Laboratory of Biomedical Imaging and Guangdong Provincial Engineering Research Center of Molecular Imaging, The Fifth Affiliated Hospital, Sun Yat-sen University, Zhuhai 519000, China

## Abstract

Most complex disease-associated loci mapped by genome-wide association studies (GWAS) are located in non-coding regions. It remains elusive which genes the associated loci regulate and in which tissues/cell types the regulation occurs. Here, we present PCGA (https://pmglab.top/pcga), a comprehensive web server for jointly estimating both associated tissues/cell types and susceptibility genes for complex phenotypes by GWAS summary statistics. The web server is built on our published method, DESE, which represents an effective method to mutually estimate driver tissues and genes by integrating GWAS summary statistics and transcriptome data. By collecting and processing extensive bulk and single-cell RNA sequencing datasets, PCGA has included expression profiles of 54 human tissues, 2,214 human cell types and 4,384 mouse cell types, which provide the basis for estimating associated tissues/cell types and genes for complex phenotypes. We develop a framework to sequentially estimate associated tissues and cell types of a complex phenotype according to their hierarchical relationships we curated. Meanwhile, we construct a phenotype-cell-gene association landscape by estimating the associated tissues/cell types and genes of 1,871 public GWASs. The association landscape is generally consistent with biological knowledge and can be searched and browsed at the PCGA website.

## Graphical Abstract

**Graphical Abstract:**
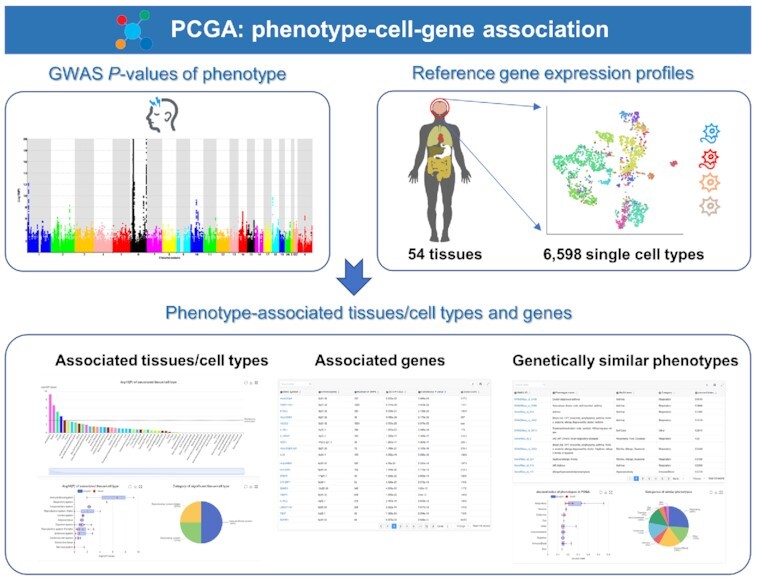
PCGA, phenotype-cell-gene association analysis platform, is a web server to jointly infer associated tissues/cell types and genes of complex diseases and traits based on GWAS summary statistics.

## INTRODUCTION

Genome-wide association studies (GWAS) have identified many variants associated with complex diseases, providing insights into the pathogenesis. However, the major (∼90%) disease-associated variants lie in the non-coding regions of the genome (1), making it challenging to translate the associated variants into the molecular mechanism underlying complex diseases. Identifying critical cell types and genes regulated by the disease-associated variants may be a primary step to elucidate etiology of complex diseases and further develope precision therapy (2). Several approaches have been developed to estimate tissues/cell types or genes associated with complex diseases by GWAS results. The methods typically integrated other omics data with GWAS results. For example, Ongen et al. estimated disease-associated tissues by enriching tissue-based eQTL in associated variants of GWAS (3). Finucane et al. estimated tissue-specific heritability enrichment of complex diseases by integrating epigenetic annotation (4). By integrating gene expression profiles of different tissues/cell types and GWAS summary statistics, deTS (5), LDSC-SEG (6), RolyPloy (7), FUMA (Cell Type function) (8) and DESE (driver tissue estimation by selective expression) (9) were developed to estimate associated tissues or cell types. In contrast to other methods, DESE can not only correctly estimate disease-associated tissues but also facilitate the prioritization of susceptibility genes (9).

The single-cell RNA sequencing (scRNA-seq) technology provides tremendous advantages for precisely profiling genes’ expression in cell types and deeply understanding cell lineage. Nowadays, scRNA-seq technology has been widely used to detect heterogeneity among tumor cells (10,11), to reveal developmental processes and cell fate decisions (12), and to profile lineages and cell types in the vertebrate brain (13). In addition, a human cell atlas project has recently provided a comprehensive human cell landscape that released gene expression profiles and cell hierarchy for over a half million cells (14). Meanwhile, several comprehensive public resources are also available to query gene expression in single cells. For instance, the PanglaoDB has collected gene expression profiles for over 1 million human cells and around 4.5 million mouse cells (15). These resources may provide a unique opportunity for deciphering the critical cell types in the development of complex diseases and traits.

Here, we expanded DESE to integrate single-cell transcriptome data and built a web server named PCGA (https://pmglab.top/pcga) to provide service for conveniently estimating associated tissues, cell types and genes by GWAS summary statistics. We collected extensive bulk RNA-seq and scRNA-seq datasets and generated gene expression profiles of 54 human tissues, 2,214 human cell types and 4,384 mouse cell types. These expression profiles provide the basis for estimating associated tissues, cell types and genes for complex phenotypes. We also analyzed 1,871 public GWASs of complex phenotypes by the PCGA analysis framework and put the association results on the PCGA web server. The associated tissues and cell types of the complex phenotypes were consistent with biological knowledge overall. We expect the web application and precomputed association resource will be widely used in deciphering the genetic mechanisms of complex diseases.

## MATERIAL AND METHODS

### Collection and process of bulk and single-cell RNA-seq dataset

We collected bulk RNA-seq datasets from GTEx projects (version 8) (16) and single-cell RNA-seq datasets from PanglaoDB (15), Human Cell Landscape (17), Allen Brain Atlas (18). For bulk RNA-seq datasets, we normalized expression values by CPM (count per million) within samples and removed batch effects by TMM (trimmed mean of M values) (19) across all samples. Then we averaged the expression values of samples in the same tissues. For single-cell RNA-seq datasets, we collected UMI (unique molecular identifier) counts matrix of single cells, cell clustering results and inferred cell-type labels for cell clusters. We filtered out the cells with < 300 UMI counts. Due to low gene abundance in single cells, we averaged UMI counts of the top 10% highly expressed cells within each cell cluster. Cell clusters with < 15 cells or unknown cell-type labels or from abnormal samples (cancer or other diseases) were removed. Cell clusters identified as the same cell types within a scRNA-seq dataset were merged. Here, we refer to each cell cluster in each dataset as a cell type. We normalized cell-type expression values by CPM. In the mouse scRNA-seq datasets, genes were mapped to their homologous human genes by the R package ‘biomaRt’ (version 2.34.2) (20). The genes were assigned with HGNC gene symbols, and genes without known HGNC gene symbols were removed. Finally, we obtained expression profiles of 54 human tissues, 2,214 human cell types and 4,384 mouse cell types. We also unified the inferred cell-type's labels and the sampling tissue/organ names of all cell types from different datasets (Supplementary Table S1).

### Collection and process of public GWAS summary statistics

We collected summary statistics of 1,871 GWASs with a large sample size (n > 10,000) and full variant records from Gene Atlas (21), GWAS Atlas (22) and Neale Lab UKBB v3 (http://www.nealelab.is/uk-biobank) according to the collection rules of CAUSALdb (23). The population information, sample size, and mapped MeSH terms were exacted from CAUSALdb. It should be noted that GWAS Atlas also collected GWAS summary statistics from the non-UKBB cohort, while these datasets actually were provided by other websites, such as GRASP (24) and PGC (https://www.med.unc.edu/pgc). Here these datasets were regarded as the GWAS datasets from GWAS Atlas. The collection details of the GWAS datasets are shown in Supplementary Table S2. We exacted the *P*-values and chromosome coordinates of all available variants. Non-GRCh37 coordinates were converted to GRCh37 coordinates, and the variants that couldn’t be converted were removed.

### PCGA analysis workflow and association landscape construction

**Figure 1.**
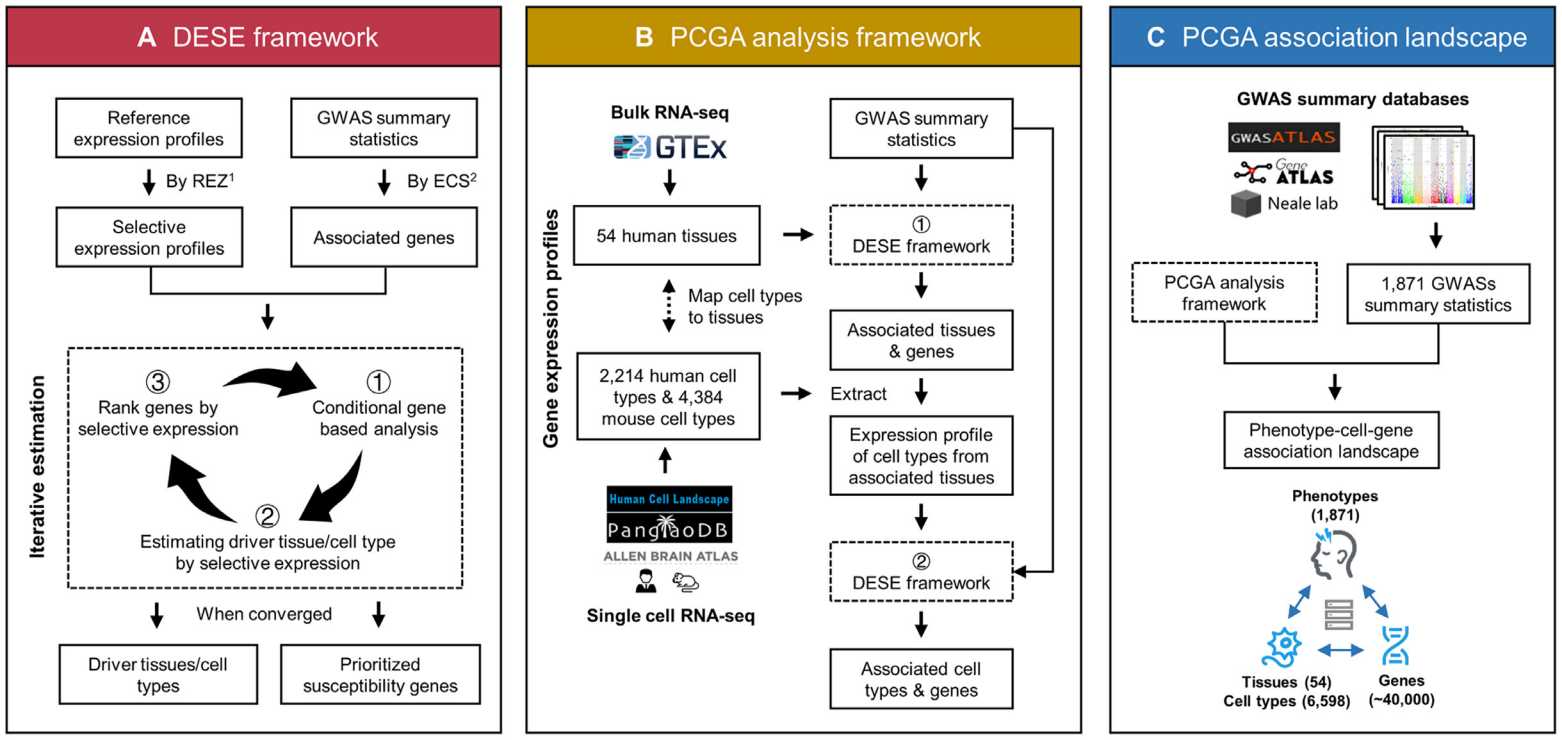
Workflow of PCGA web server. (**A**) Workflow of DESE framework (9). REZ, robust regression Z-score, a method to calculate tissue selective expression (9). ECS, effective chi-square, a gene-based analysis method (25). (**B**) Workflow of PCGA analysis framework. (**C**) Workflow to construct PCGA association landscape.

The core method of PCGA to estimate associated tissues/cell types and genes is based on DESE (9) (driver tissue estimation by selective expression), which was proposed by our group in 2019. DESE estimates driver tissues and susceptibility genes by integrating GWAS summary statistics and tissue expression profiles. The underlying assumption is that phenotype-associated genes tend to be selectively expressed in driver tissues of phenotype. The driver tissues are estimated by testing the higher selective expression of phenotype-associated genes in a tissue or cell type. Meanwhile, the estimation of phenotype-associated genes can be promoted by adding selective expression information in an iterative procedure. The main steps in estimation are described below (Figure 1A). First, the associated genes are estimated by ECS (effective chi-square) (25) with GWAS *P*-values of phenotype. In this step, the genotypes of the ancestrally matched panel of the 1000 Genomes Project (26) with the input GWAS samples are used to calculate linkage disequilibrium (LD) coefficients, which are then employed to remove redundant associations among variants by the ECS (25). Second, the selective expression profiles of tissues/cell types are calculated by REZ (robust regression Z-score) (9). Thirdly, the associated genes and selective expression profiles were inputted into a model, where the conditional gene-based analysis and associated tissues/cell-types estimation were iteratively carried out to output converged results. The associated tissues/cell types are estimated according to selective expression enrichment of phenotype-associated genes by the Wilcoxon rank-sum test. The conditional gene-based analysis was guided by the selective expression of genes in the associated tissues/cell types.

PCGA uses a hierarchical estimation strategy to estimate associated tissues and cell types sequentially. Firstly, the associated tissues are detected by DESE with bulk RNA-seq based reference expression profiles. Then PCGA extracts scRNA-seq based reference expression profile of cell types belonging to associated tissues to estimate associated cell types by DESE again (Figure 1B). We mapped the cell types of scRNA-seq datasets and tissues of bulk RNA-seq datasets to 43 unified organs/tissues according to their sampling tissues/organs (Supplementary Table S1). Therefore, PCGA can automatically extract the expression profiles of cell types belonging to the associated tissues. Meanwhile, PCGA also allows users to manually select expression profiles of cell types by the unified organs/tissues based on their prior knowledge of the target phenotypes. The associated genes are also prioritized in two estimation steps above.

We estimated associated tissues, cell types and genes of 1,871 public GWASs by the above workflow to construct a phenotype-cell-gene association landscape. The association landscape can be searched and visualized in the PCGA web server (Figure 1C).

### Input

**Figure 2.**
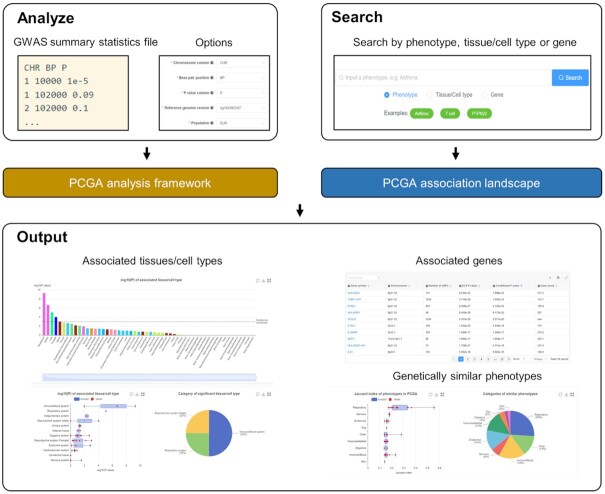
Input and output of PCGA web server. PCGA allows analyzing the user's uploaded GWAS summary statistics file and searching the precomputed association landscape.

In the PCGA analysis function, users should upload a GWAS summary statistics file firstly (Figure 2). The file should be a tab- or comma-delimited text file containing at least three columns, i.e. chromosome number, base pair position (based on hg19/38) and *P*-value. Each line represents a variant, and the header line is required. It should be noted that the variants in the GWAS summary file must be full because the gene-based association test in PCGA cannot use the pre-selected variants according to a *P*-value threshold. The usage of significant variants will inflate the false-positive rates of the gene-based test and lead to an unreliable inference of associated cell types. After the file is uploaded successfully, users should fill in several job options. In this step, the users can select reference expression profiles of tissues/cell types. In addition, users should select an ancestrally matched panel of the 1000 Genomes Project with the input GWAS sample to ensure that the gene-based association analysis can be performed correctly. The details of the options are explained on the webpage. Once the job is submitted successfully, the user will be assigned a unique link to check the progress and results of the job.

PCGA also allows users to access precomputed phenotype-cell-gene association landscape by searching keywords of phenotypes, tissues/cell types and genes or browsing the categories’ tree of phenotypes and tissue/cell types (Figure 2).

### Output

In the PCGA analysis function, the associated tissues, cell types and genes are returned to users. The strength of the association is mainly measured by the *P*-values. The results are presented in interactive figures and tables, which can be downloaded directly. In addition, PCGA provides the genetic similarities between the user's phenotype and 1,871 precomputed phenotypes. The genetic similarity is measured by the Jaccard similarity coefficient of significantly associated genes. The genetic similarity can help users recognize the genetic relationship and pathogenic mechanism similarity between uploaded GWAS and public GWASs (Figure 2).

A similar output will be returned if the user retrieves precomputed association landscape. The associated tissues/cell types and genes will be returned when searching for a phenotype. The selective expression profiles of tissues/cell types and associated phenotypes will be returned when searching for a gene. When searching for a tissue/cell type, the selectively expressed genes and associated phenotypes will be returned.

### Web server implementation

**Figure 3.**
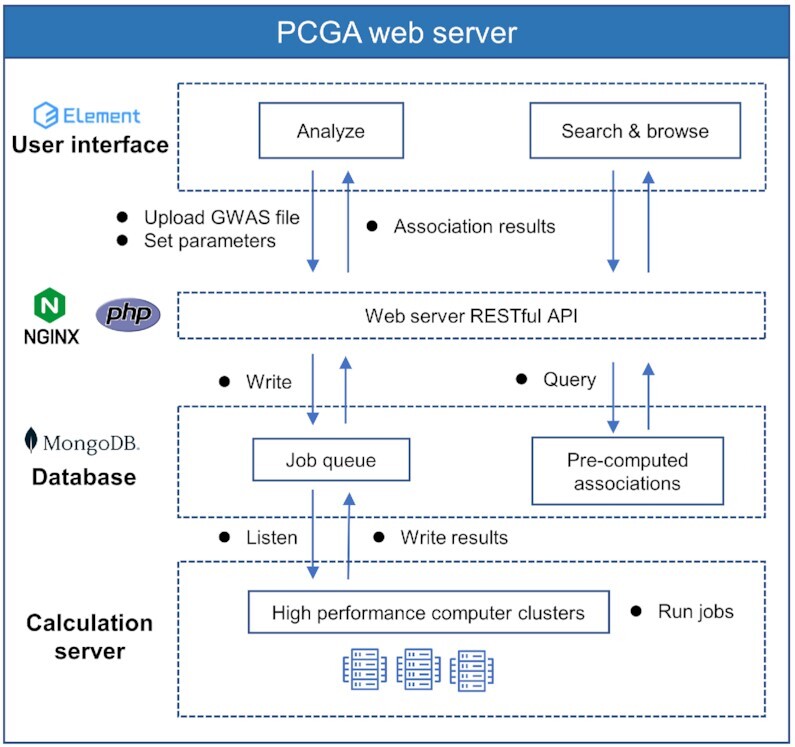
Web server implementation.

The PCGA web server adopts the Representational State Transfer (REST) (27) design style to separate the front-end and back-end designs. The front-end is responsible for friendly interface display, and the back-end is responsible for business logic (Figure 3). We use a web user interface (UI) framework on the front-end called Vue (version: 2, https://vuejs.org) for development, and most of the webpages are developed with the Element UI library (version: 2.15.7, https://element.eleme.io/). The vxe-table library (version: 3.4.10, https://github.com/x-extends/vxe-table) is used to render tables, and the ECharts library (Version: 4.8.0, https://echarts.apache.org) is used to generate figures. In the back-end, we use PHP (version: 7.3.23, https://www.php.net) to write the web application programming interface (API). We use a non-relational database MongoDB (version 4.4.10, https://www.mongodb.com) to store data resources and job information. We run a program on a high-performance computing cluster (HPC) to monitor the un-running jobs in the queue database. The main program for calculating phenotype-associated tissues, cell types and genes in the back-end is in KGGSEE (https://pmglab.top/kggsee). The container of the web server is Nginx (version: 1.18.0, https://www.nginx.com).

### Global analysis of phenome associated tissues and cell-types

To explore the associated tissues and cell types of 1,871 phenotypes globally, we test the enrichment between phenotype category and tissue/cell-type category. According to the clinical characters, the phenotypes are manually classified into 19 categories (see details in Supplementary Table S2). The tissues are classified into 12 categories, and the cell types are classified into six categories (see details in Supplementary Table S1). Then we calculated enrichment *P*-values between phenotype categories and tissue/cell-type categories by hypergeometric test. Assumed that we obtained a total of *N* association *P*-values, of which *M* association *P*-values are significant (FDR-adjusted *P*-value < 0.05). There are *n* association *P*-values between phenotype category *H* and tissue/cell-type category *T*, of which *k* reach the significance level. Then the enrichment *P*-value for phenotype category *H* and tissue/cell-type category *T* is:

$$\begin{equation*}{\rm{\;}}{P_{HT}} = \;1 - \mathop \sum \limits_{x\; = \;0}^{k - 1} \frac{{\left( {\begin{array}{@{}*{1}{c}@{}} M\\ x \end{array}} \right) \times \left( {\begin{array}{@{}*{1}{c}@{}} {N - M}\\ {n - x} \end{array}} \right)}}{{\left( {\begin{array}{@{}*{1}{c}@{}} N\\ n \end{array}} \right)}}\end{equation*}$$

### Comparison to FUMA Cell Type

Watanabe et al. proposed a similar approach to estimate associated cell types by GWAS summary statistics and expression profiles of cell types, implemented in the web server FUMA Cell Type (a subfunction of FUMA, https://fuma.ctglab.nl) (8). For simplicity, FUMA Cell Type is hereinafter referred to as FUMA. We compared PCGA with FUMA in terms of method principle, data resources, and web server characteristics. We also compared their performance in estimating associated cell types of three representative complex diseases, i.e. coronary artery disease, major depression and rheumatoid arthritis. The GWAS summary statistics of three complex diseases are up-to-date (see details in Supplementary Table S3). In the first comparison, we used the same expression dataset to compare the performance of core methods underlying PCGA and FUMA. We select a dataset generated by FUMA, i.e. the Tabula Muris FACS (28) expression dataset, including 119 cell types (https://github.com/Kyoko-wtnb/FUMA_scRNA_data/blob/master/processed_data/TabulaMuris_FACS_all.txt.gz), which was used for comparing to other methods in the FUMA Cell Type paper (8). In the second comparison, we used respective expression datasets included in PCGA and FUMA web servers for comparison. For PCGA, the expression profiles of cell types are automatically selected based on associated tissues results. All of the unique cell-type expression datasets are selected to run for FUMA. Bonferroni-adjusted *P*< 0.05 is used to define significant association.

## RESULTS

### Global analysis of phenome associated tissues and cell-types

**Figure 4.**
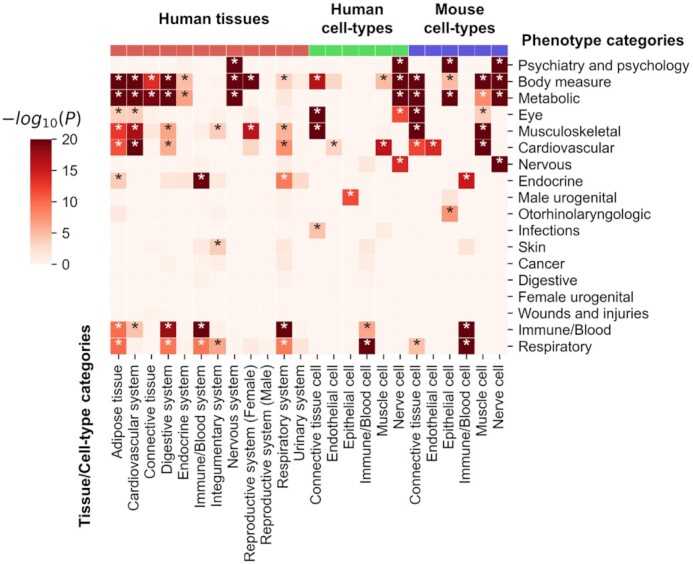
Enrichment *P*-values between phenotype categories and tissue/cell-type categories. The horizontal axis of the heatmap represents tissue/cell-type categories. The color bars above the heatmap represent transcriptome data types (the red bar represents human tissues, the green bar represents human cell types, and the blue bar represents mouse cell types). The vertical axis represents the phenotype categories. The heatmap colors indicate -log_10_(*P*-value), and asterisks indicate Bonferroni-adjusted *P*< 0.05. To improve visualization, *P*-values are thresholded at 10^–20^.

We overviewed the phenome-associated tissues and cell types globally by testing the enrichment between phenotype category and tissue/cell-type category (Figure 4, Supplementary Table S4). Most of the associations are consistent with known biology. At the tissue level, psychiatry and psychology phenotypes are significantly (Bonferroni corrected *P*< 0.05) associated with nervous system tissues (*P*= 2.24 × 10^–242^). The most significantly associated tissues of cardiovascular phenotypes are cardiovascular system tissues (*P*= 4.70 × 10^–23^). Immune/blood phenotypes are significantly associated with immune/blood tissues (*P*= 1.95 × 10^–62^). In addition, body measurement (including height, BMI, etc.) and metabolism-related phenotypes were significantly associated with a wide range of tissues, including adipose tissue, cardiovascular system, connective tissue and nervous system. Respiratory system phenotypes are significantly associated not only with respiratory system tissues (*P*= 8.89 × 10^–10^) but also with immune/blood tissues (*P*= 4.90 × 10^–10^), which may be because a good part of respiratory phenotype is related to immunity (e.g. asthma and rhinitis). At the human cell-type level, 15 phenotype category-cell type category associations are significant, of which 12 are also significant in the mouse cell-type dataset. Similar to the tissue-level association results, most phenotype-cell type associations are consistent with known biology. For example, in both human and mouse datasets, psychiatry and psychology phenotypes are significantly associated with nerve cells (*P* = 0). The cardiovascular phenotypes are significantly associated with endothelial cells (human *P*= 3.96 × 10^–4^, mouse *P*= 1.21 × 10^–14^) and muscle cells (human *P*= 1.47 × 10^–16^, mouse *P*= 1.70 × 10^–36^). Immune/blood phenotypes are significantly associated with immune/blood cells (human *P*= 4.35 × 10^–7^, mouse *P*= 7.22 × 10^–168^). Constant with tissue-level results, body measurement-related phenotypes are significantly associated with multiple categories of cell types, i.e. nerve cells (*P*= 0 in both human and mouse datasets), connectivity tissue cells (human *P*= 4.68 × 10^–17^, mouse *P*= 0) and muscle cells (human *P*= 1.07 × 10^–5^, mouse *P*= 3.39 × 10^–36^). In addition, respiratory phenotypes are significantly associated with immune/blood cells (human *P*= 1.64 × 10^–23^, mouse *P*= 1.78 × 10^–107^), which is consistent with the tissue-level findings, suggesting the key role of immunity in most respiratory phenotypes. In summary, the above results suggested PCGA analysis framework could effectively estimate the associated tissues and cell types for complex phenotypes overall.

### Case studies

We use the GWAS of asthma (22) as an example to show the analysis function of PCGA (see results in https://pmglab.top/pcga/#/results/phenotype_task?task_id=edd59e0b827974dc64a755d46dc1c59f). Whole blood (*P*= 4.8 × 10^–10^), spleen (*P*= 2 × 10^–7^) and lung (*P*= 8.3 × 10^–6^) are estimated as the top three significant tissues associated with asthma. T cell is estimated to be the most significant cell type associated with asthma in both human (*P*= 6.6 × 10^–9^) and mouse (*P*= 5 × 10^–6^) datasets. Many studies have shown that asthma is related to various T cell types (29,30). PCGA estimated 118 genes as the significant susceptibility genes of asthma (FDR corrected *P*< 0.05). The top 50 similar phenotypes of asthma mainly include asthma from other GWASs, allergic rhinitis, rheumatoid arthritis, type 1 diabetes, thyrotoxicosis, etc. Although these diseases occur in different parts of the human body, they are all related to immunity (31–35).

We show an example of accessing the PCGA association landscape by searching the gene *PTPN22* (see the searching results in https://pmglab.top/pcga/#/results/gene?id=PTPN22). The top three significant tissues selectively expressing *PTPN22* are immune, i.e. EBV-transformed lymphocytes, whole blood and spleen. At the cell type level, *PTPN22* is selectively expressed in T cells and NK cells in both human and mouse datasets. The associated phenotypes of *PTPN22* mainly include thyroid diseases, rheumatoid arthritis and diabetes, which are relevant to immune abnormalities (34,35). The above association results suggest *PTPN22* plays an important role in maintaining normal immunity.

### Compare to FUMA

FUMA web server also allows estimating associated cell-types of complex diseases by GWAS summary statistics, but PCGA and FUMA are different (Table 1). In the principle of the method, PCGA is based on an iterative estimation framework DESE, which subtly allows the estimation of associated cell types and prioritization of susceptibility genes to help each other. FUMA estimates associated cell types by a regression model with associated genes produced by MAGMA (36). In terms of expression resources, the PCGA web server includes more cell types (6,598) than FUMA (2,679). PCGA also integrates bulk RNA-seq datasets of 54 human tissues to estimate associated tissues. Moreover, the cell type labels and sampling tissue/organ labels of expression profiles in PCGA are manually unified, making it easy for users to understand the meaning of cell types. In contrast, the expression profiles in FUMA are based on raw cell type labels provided by corresponding studies with different naming standards, making it difficult to understand the meaning of the cell type labels in some cases. Regarding the selection of cell-type expression profiles, PCGA allows automatically selecting cell types by associated tissues results or manually selecting by unified tissues/organs. FUMA only allows selecting datasets manually. Most importantly, PCGA provides an association landscape among phenotypes, tissues/cell types and genes by analyzing 1,871 public GWASs. Below we also compared the performance of PCGA and FUMA in estimating associated cell types of three complex diseases.

**Table 1.** General comparison of PCGA and FUMA

	PCGA	FUMA
**Core Method**	DESE, subtly allows the estimation of associated cell types and prioritization of susceptibility genes to help each other	Regression model based on associated genes of MAGMA and expression profile
**Expression Dataset**	6,598 cell types (human and mouse) and 54 human tissues Manually unified cell type labels and sampling tissue/organ labels	2,679 cell types (human and mouse) Raw cell type labels
**Selection of cell types’ expression profile**	(1) Automatically select by associated tissues results (2) Manually select by unified tissues/organs	Manually select by datasets
**Phenotype-cell-gene association landscape**	Associated tissues, cell types and genes of 1,871 public GWASs	No

**Figure 5.**
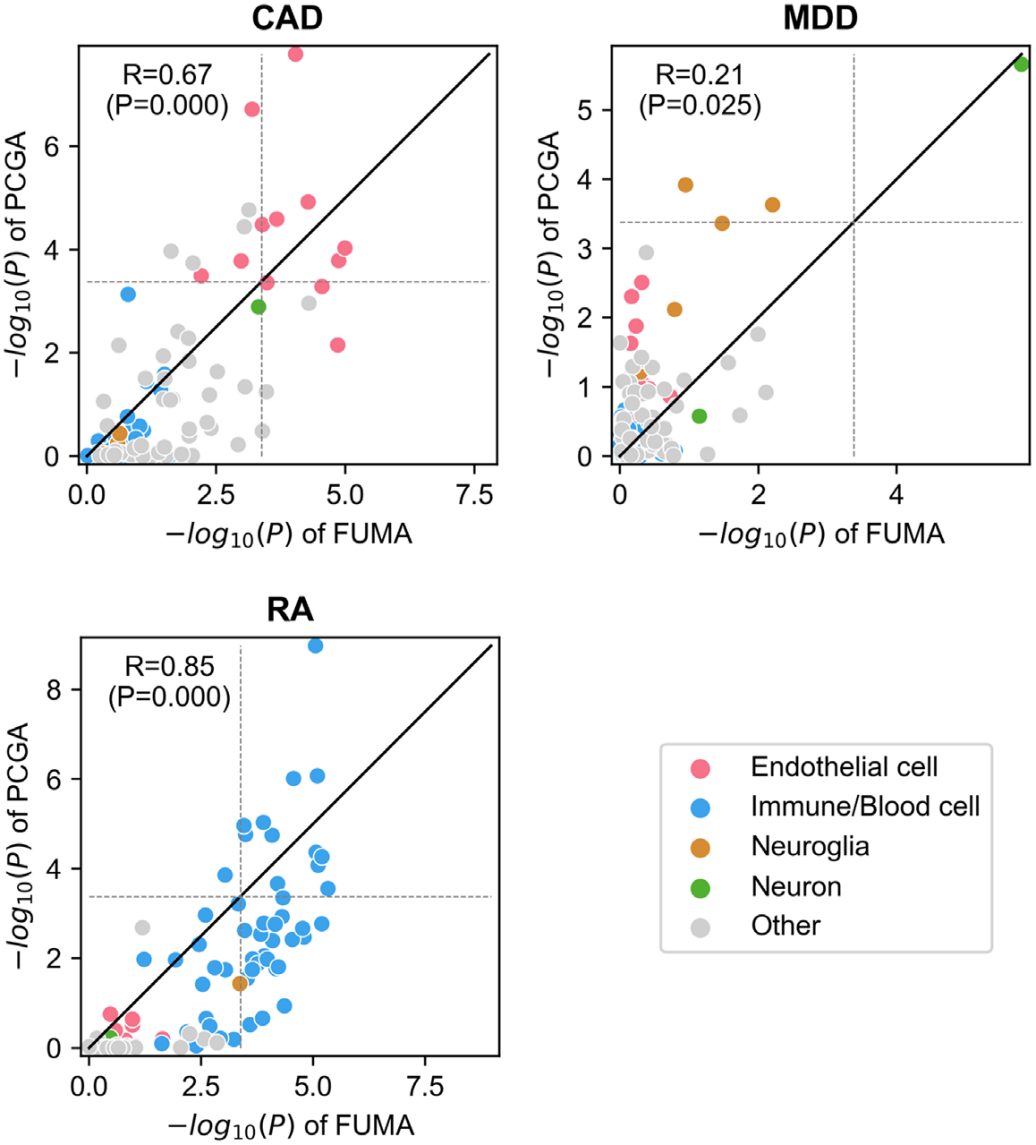
*P*-values of associated cell-types estimated by PCGA and FUMA with same expression dataset in three complex diseases. CAD, coronary artery disease. MDD, major depression. RA, rheumatoid arthritis. The horizontal axis represents -log_10_(*P*) of FUMA and the vertical axis represents -log_10_(*P*) of PCGA. Each dot represents a cell type, and the dot's color indicates the cell type category. The solid black line represents *y*= *x*. The dashed grey lines represent the significance threshold (Bonferroni-adjusted *P*< 0.05). The correlation of the *P*-values between PCGA and FUMA is measured by Spearman's correlation coefficient.

In the comparison using the same expression dataset, although the estimation results of PCGA and FUMA are similar overall (Figure 5, Supplementary Table S5-7), PCGA shows greater sensitivity and specificity in some cases. The Spearman's correlation coefficients of *P*-values of associated cell types between PCGA and FUMA are 0.67, 0.21, 0.85 in coronary artery disease (CAD), major depression (MDD) and rheumatoid arthritis (RA), respectively, and the correlations are significant (*P*< 0.05). PCGA estimated 13 significantly (Bonferroni adjusted *P*< 0.05) associated cell types for CAD, of which nine cell types are endothelial cells. Similarly, FUMA estimated 12 significantly associated cell types for CAD, with nine endothelial cells. Six endothelial cell types are significant in both PCGA and FUMA. Many studies have reported the important roles of endothelial cells in the occurrence and development of CAD (37,38). Interestingly, the most significant cell type estimated by PCGA is endothelial cells of the heart (*P* = 1.64 × 10^–8^, Supplementary Table S5). The most significant cell type estimated by FUMA is endothelial cells of the trachea (*P*= 1.014 × 10^–5^). Based on prior knowledge, endothelial cells of the heart may be more relevant to CAD than endothelial cells of the trachea. For MDD, both PCGA and FUMA estimated brain neurons as the most significantly associated cell type with similar *P*-values (*P*_PCGA_= 2.20 × 10^–6^, *P*_FUMA_= 1.57 × 10^–6^). In addition, PCGA specifically estimated two neuroglia cell types as the significant cell types, i.e. astrocyte (*P* = 1.21 × 10^–4^) and oligodendrocyte precursor cell (*P* = 2.35 × 10^–4^). Several literatures also indicate the important role of neuroglia cells in the development of MDD (39,40). These studies highlight the involvement of neuroglia cells in the process of neuroplasticity through signaling and immunity, suggesting the important role of glial cells in the pathophysiology of depression and the development of antidepressants. For RA, both PCGA and FUMA estimated 12 significant immune cell types, mainly including B cells, T cells and NK cells from different body regions. Many studies have indicated the role of B cells, T cells and NK cells in the development of RA (41–44). Marrow B cell is estimated as the most significant cell type by PCGA (*P* = 1.06 × 10^–9^), and marrow immature T cell is estimated as the most significant cell type by FUMA (*P* = 4.66 × 10^–6^).

In the comparison using respective expression datasets of PCGA and FUMA, the main estimation results are consistent (Table 2, Supplementary Table S8-9). However, the associated cell types estimated by PCGA were more refined in some cases. Both two web servers estimated endothelial cells and smooth muscle cells as significantly associated cell types of CAD (Bonferroni adjusted *P* < 0.05), which are proved by many kinds of literature (37,38,45). Interestingly, PCGA also estimated fibroblasts as the associated cell type for CAD, which is reported to play an important role in atherosclerosis (46). For MDD, both PCGA and FUMA estimated neurons as associated cell types. PCGA estimated three glial cell types, i.e. astrocytes, microglias and oligodendrocytes, as associated cell types of MDD. FUMA estimated one glial cell type, ependymal cells, as an associated cell type of MDD. Associated cell types of RA estimated by both PCGA and FUMA are immune/blood cells. However, the associated cell types estimated by FUMA are relatively rough, such as professional antigen-presenting cells, leukocytes, and blood cells. By contrast, PCGA estimated more refined immune cell types as associated cell types for RA, such as B cells, T cells, NK cells, dendritic cells, monocytes, mast cells and macrophages.

**Table 2.** Significantly associated cell types of three complex diseases estimated by PCGA and FUMA with respective expression datasets (Bonferroni adjusted *P* < 0.05). CAD, coronary artery disease. MDD, major depression. RA, rheumatoid arthritis

	PCGA	FUMA
**CAD**	Endothelial cells, smooth muscle cells, fibroblasts, stellate cells (quiescent fibroblasts)	Endothelial cells, mural cells (containing vascular smooth muscle cells), leukocytes
**MDD**	Neurons, astrocytes, microglias, oligodendrocytes	Neurons, ependymal cells
**RA**	Lymphocytes, T cells, B cells, NK cells, dendritic cells, monocytes, mast cells, macrophages	Professional antigen-presenting cells, leukocytes, blood cells, macrophages, microglias

## DISCUSSION

The PCGA web server provides a unified framework for jointly estimating associated tissues, cell types, and genes of complex diseases and traits by GWAS summary statistics. It has extensive expression profiles of 54 tissues and 6,598 cell types to support efficiently estimating associated tissues and cell types for complex phenotypes. By analyzing 1,871 public GWASs, we build a comprehensive phenotype-cell-gene association landscape and put it on the PCGA webserver to share with researchers. We also showed that the associations are consistent with known biology overall, suggesting that the PCGA framework is robust and reasonable. As far as we know, the association landscape is a resource for presenting phenome-associated cell types for the first time. We expect the association landscape to be useful for annotating complex phenotypes, tissues/cell types, and genes. Compared to a similar web server FUMA (8) in estimating associated cell types for three complex diseases, we showed that PCGA is generally consistent with it. At the same time, we noticed that PCGA could keenly find more reasonable phenotype-associated cell types. For example, PCGA specifically estimated endothelial cells from the heart rather than other organs as the most significant cell type associated with coronary artery disease. Moreover, PCGA uniquely estimated two types of neuroglia cells as significant cell types associated with major depression, which was supported by several works of literature (39,40). This may be because the core method of PCGA, DESE, performs selective expression-guided conditional gene association analysis to remove the redundant associated genes. Regarding expression resources, PCGA also provides more cell types than FUMA and offers unified cell type labels to make it easier for users to understand the meaning of cell types.

The basic assumption of PCGA’s core method, DESE, is that the phenotype-associated genes (regardless of their directions) tend to be high-selectively expressed in driver tissues/cell types in normal (or healthy) samples. PCGA only requires the input of GWAS *P*-values of variants, making the analyses of associated genes and associated tissue/cell types very convenient. Although PCGA does not consider the direction of the associated genes, our analysis results show that it can accurately estimate the associated genes and tissue/cell types of complex phenotypes.

In summary, the PCGA web server provides an online tool and a comprehensive resource to easily explore associations between complex phenotypes, tissues/cell types, and genes. We will continue to expand the PCGA web server to provide more functions to parse GWAS signals of complex phenotypes. For example, we can use Mendelian randomization methods to recognize the causal gene by integrating multiple levels of molecular traits quantitative loci data, such as eQTL and sQTL.

## DATA AVAILABILITY

The web server is freely available at https://pmglab.top/pcga. The expression data and phenotype-cell-gene association data are available at https://pmglab.top/pcga/#/download.

## Supplementary Material

gkac425_Supplemental_File
